# Mapping of dental graduates’ career paths in Hong Kong, Japan and mainland China

**DOI:** 10.3389/froh.2022.994613

**Published:** 2022-11-03

**Authors:** Chloe Meng Jiang, Takashi Nishioka, Guang Hong, Hao Yu, Chang-yuan Zhang, Chun Hung Chu

**Affiliations:** ^1^ Faculty of Dentistry, The University of Hong Kong, Hong Kong SAR, China; ^2^ Liaison Center for Innovative Dentistry, Graduate School of Dentistry, Tohoku University, Sendai, Japan; ^3^ School and Hospital of Stomatology, Fujian Medical University, Fuzhou, China

**Keywords:** dental graduate, dental student, career path, career development, dentist

## Abstract

Dental graduates have a variety of career-path choices. After graduation, they may join private dental practice, government- or hospital-based dental care services, research groups, academia, business or industry. With globalization and frequent international exchange, dental graduates nowadays can explore careers outside their home country. However, dental education systems and job opportunities vary widely across different regions and countries. Diversity of accreditation in dental education, different licensure requirements, and lack of global competencies in dental care often limit the globalization, operation and survival of dental practice and education worldwide. The requirements for professional education and practice can be quite diverse, and these differences will be barriers to dental graduates seeking career development outside their home home country. Fresh dental graduates have minimal experience in job hunting. More specifically, they are unfamiliar with potential career paths. This paper was based on the 4th trilateral symposium 2022 organized by The University of Hong Kong, Tohoku University, and Fujian Medical University, which offered a lecture to discuss career paths for dental graduates in Hong Kong, Japan, and mainland China. The aim of this paper was to provide dentists, particularly fresh graduated dental students, with practical insight into different career paths in Hong Kong (Special Administrative Region of China, SAR), Japan and mainland China, and factors that may influence their career options. It assists dental students in exploring possibilities in dentistry and preparing for their career development after graduation from dental school.

## Introduction

Fresh dental graduates may find themselves pondering questions when it comes to graduation. After years of learning and training in dental school, they are very experienced as students, but they are indeed rookies in finding career tracks. In this paper, we refer to “dental graduates” as students who have just finished undergraduate study and have obtained the dental degree. There are many choices for dental graduates in deciding their career paths. They can practice dentistry in private sectors, in large dental groups, or in government- or hospital-based dental care facilities. Other than jumping into the job market, dental graduates can apply for advanced training in a clinical specialty or in academic research. Dental graduates who are not interested in practicing as clinicians may go into business or industry. However, not all dental graduates are fully aware of their career options when it comes to graduation (1).

Dental graduates in different places report a variety of motivations and plans for career development. In mainland China, according to a survey conducted in 2022, a majority of dental graduates (88%) decided to pursue postgraduate degrees to increase their competitiveness in finding better jobs (2). Three favourite disciplines ranked by students were prosthodontics, oral surgery, and orthodontics (3). However, in Japan, the majority of dental graduates (57%) wanted to be general practitioners working at private dental clinics, while only 26% of students wished to be specialists (4). Likewise, in the United Kingdom, a cross-sectional survey showed that around half of final-year dental students hesitated to pursue specialist careers, while approximately 38% wished to specialize in one discipline (5). Three favourite disciplines selected by them were restorative dentistry, orthodontics and oral surgery. In Hong Kong (Special Administrative Region of China, SAR), the majority of dental graduates work as general practitioners in private sectors, and only a few of them wish to pursue specialty training (6).

With the fast pace of globalization, dental graduates can seek career development within as well as outside their home country. There is an emerging phenomenon of international migration of dentists, which has been named “global interconnectedness” (7). Complex reasons were indicated for the origins of desires to migrate, which involves social and political issues, and an historical aspect stimulated by priori knowledge and interactions with people, place and things (7). However, due to variations of dental education systems and job opportunities in different places, dental graduates may not fully understand the situation outside their home country or the place they received dental training (8).

In the literature, Mariño et al. (9) presented an overview of the career paths currently open to oral health professionals, covering paths in general dentistry (in private practices, community centers, and hospitals) and each of the various specialties, academic related career paths in both teaching and research, and non-traditional paths (community, government, administration, policy making, government research, and oral health organizations). In the present paper, we would like to focus on the career development in the specific places, i.e., Hong Kong, Japan and mainland China, where health care systems and dental education systems are different. *The 4th trilateral symposium 2022* organized by The University of Hong Kong, Tohoku University, and Fujian Medical University offered a lecture to discuss career paths for dental graduates in Asia, i.e., Hong Kong, Japan, and mainland China (by alphabetic order). This paper is a summary of the lecture. The present paper can provide local dental students with practical insight into different career paths, and also shine a light on international dentists who are interested in migrating to the above-mentioned places.

The aim of the paper is to provide dentists, particularly fresh graduated dental students, with practical insight into career paths in Hong Kong, Japan, and mainland China, and factors that may influence their career options. This paper will assist dental students consider their choices for future professional development, or acquaint themselves with issues and debates.

## Material and method

**Figure 1:**
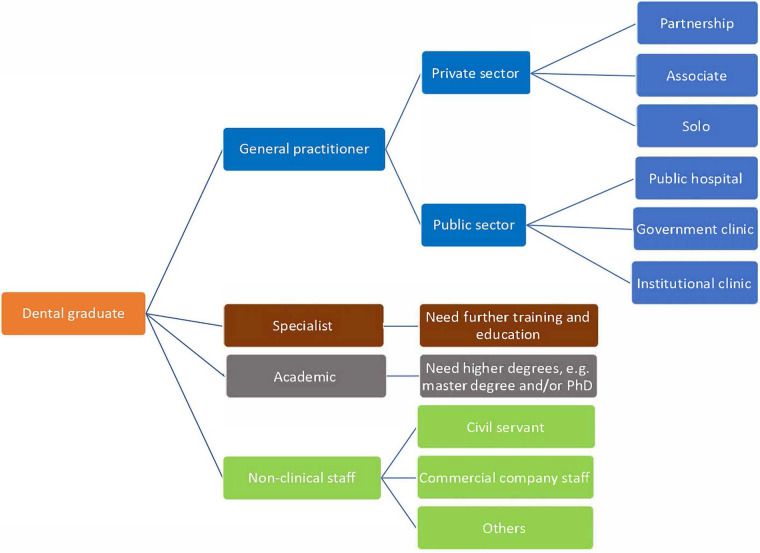
Career paths that dental graduates can pursue.

Authors from Hong Kong, Japan, and mainland China were invited to contribute to this paper. Local dental education systems were described generally. Different career paths and options such as working as a dental clinician or pursuing postgraduate training were presented (Table 1 and Figure 1). In addition, job opportunities for dental graduates to work as a researcher in academia or to conduct non-clinical activities were discussed.

**Table 1:** Summary of career paths for dental graduates.

Category	Job description	Location	Remarks
Private dentist	• Work as solo or in group (in partnership) practice • Provide comprehensive dental care for the public	Private clinic	• High flexibility • Freedom in dental practice • Owner or partner of dental clinic • Commission based remuneration
Dentist (employed)	• Provide comprehensive dental care for the public	Institutional clinic	• General dental practice • Fixed salary or depends on location
Government dentist^a^	• Provide dental care for civil servants and their dependents; • Provide dental care for primary school pupils • Promote oral health for the public	Governmental dental clinic	• Government employee • Fixed salary • Opportunity for advanced training • Guidance from senior dentists
Hospital dentist	• Provide dental care for hospitalised patients and outpatients • Work in collaboration with a medical team	General hospital^#^; Dental hospital affiliated with dental school	• Fixed salary or depends on locations • Opportunity for advanced training
Civil servant^b^	• Engaged in service work in various state agencies	Government	• Stable job • high social status
Dental company consultant	• Serve as production technicians, managers, or sales of dental materials and equipment companies	Commercial company	• Good benefits • Broad perspective
Academics	• Teach and train dental students • Conduct dental research	University, college	• Summer/winter vacations • Stable income

The category in the table can be applied to the three places, (i.e. Hong Kong, Japan and mainland China), except for those with * and #.

^a^ The category is applied to Hong Kong.

^b^ The category is applied to Japan and mainland China.

## Main text (by alphabetical order)

### Hong Kong

The Faculty of Dentistry of the University of Hong Kong (HKU) is the only dental school in Hong Kong. Graduates from the Faculty of Dentistry receive the degree of Bachelor of Dental Surgery (BDS). At present, a six-year BDS curriculum encompassing both compulsory “University Requirements” and a “Professional Core” is provided to relevant students (10). In 2020, HKU had 402 BDS students and 1,848 BDS alumni (11) who constitute the majority of dentists in Hong Kong. The Dental Council of Hong Kong recognizes the HKU BDS degree, and upon registration HKU dental graduates can practice dentistry in Hong Kong without a licensing examination.

Dental graduates trained outside Hong Kong must pass the licensing examination held by the Dental Council of Hong Kong before they can practice dentistry in Hong Kong (12). The licensing examination is held twice a year, and the first sitting is usually held from April to July while the second sitting is from September to December. The licensing examination consists of three separate parts and all three parts are held in Hong Kong. Part I is a written test of multiple-choice-questions relating to 8 subjects including basic science and various dental disciplines. Part II is practical test designed to test candidates’ manual dexterity and professional competence. Part III is to test the candidate’s ability to apply professional knowledge in clinical scenarios particularly in diagnosis, treatment planning and patient management. Details of the licensing examination can be found on the website of the Dental Council of Hong Kong (https://www.dchk.org.hk).

In general, HKU dental graduates can secure gainful employment due to a low dentist-to- population ratio (1:3000) and a high demand for dental care in the local community. Since dental care services in Hong Kong are principally provided by private sectors, most dental graduates will join and practice dentistry in private sectors. They can also join the Department of Health as government dentists. A few of them work as junior hospital dental officers at the university hospital (Prince Philip Dental Hospital) for one year before enrolling in a full-time master degree program for advanced training.

Dental care services in Hong Kong are mainly provided by private sectors under a free-market economic model (13). Dental graduates choosing this career path have plenty of flexibility and opportunity. They can provide a full range of dental treatments to their patients. Regarding the working mode, they can choose to practice solo or in partnership with other dentists. In addition, they can join various organizations, including non-government organizations as employed dentists. The service they provide is generally basic dental care to address the large dental demands of the community. In addition, dental graduates can also choose to practice in government clinics or hospitals. Through the Department of Health, the government employs more than 300 dentists, about 10% of dentists in Hong Kong (14). Government dentists provide comprehensive dental services for civil servants and their dependents. They also supervise dental therapists to provide services to primary school pupils in governmental school dental clinics. Some government dentists work in general hospitals to provide treatment for in-hospital patients. Moreover, government dental officers in the Oral Health Education Unit work to promote education to the public on dental care. Apart from these, a few dental graduates stay and work as junior hospital dental officers in the Prince Philip Dental Hospital which is associated with HKU. They work in the post for one year, after which they can apply for advanced clinical training and pursue a master’s degree.

The HKU Faculty of Dentistry offers various postgraduate degrees and diploma courses (both full-time and part-time) for students pursuing further study and training in dentistry (15) (Table 2). Courses for the Master of Dental Surgery (MDS) degree include major dental disciplines, such as endodontics, paediatrics, periodontology, prosthodontics and oral and maxillofacial surgery. Dentists who complete three-year master’s degrees can continue their advanced training to be specialists. A Master of Science in community dentistry, dental material science and implant dentistry as well as an advanced diploma in orthodontics are also available. The courses provided by the HKU Faculty of Dentistry is self-funded, but the government offers subsidies to dental graduates who are permanent Hong Kong residents. These courses are set up for dental undergraduates who want to improve their dental skills and knowledge and aspire to become dental specialists. A dental specialist in Hong Kong needs six years of postgraduate training. Specialist training generally consists of one year of fulltime general dental practice, three years of fulltime MDS training and two years of supervised higher training. However, some disciplines such as paediatric dentistry and oral and maxillofacial surgery can consist of six years of in-house government training. A dental specialist in community dentistry requires one year of full-time general dental practice, one year of full-time MSc training and four years of supervised higher training.

**Table 2:** Options for further study and training for dental graduates.

Program	Duration (mode)	Discipline
**Hong Kong**		
Master of Dental Surgery (MDS)	3 years (full-time)	• Endodontics • Paediatric Dentistry • Periodontology • Prosthodontics • Orthodontics & Dentofacial Orthopaedics • Oral & Maxillofacial Surgery
Master of Science (MSc)	1 year (full-time) 2 years (part-time)	• Community Dentistry • Dental Materials Science • Implant Dentistry • Oral & Maxillofacial Radiology & Diagnostic Imaging
Advanced Diploma in Dental Surgery (AdvDipDS)	2 years (part-time)	• Orthodontics
Master of Philosophy (MPhil)	2 years (full-time) 3 years (part-time)	• Fields related to Dentistry
Doctor of Philosophy (PhD)	4 years (full-time) 6 years (part-time)	• Fields related to Dentistry
**Japan**		
Doctor of Philosophy (PhD)	4 years (full-time)	• Fields related to Dentistry
**Mainland China**		
Postgraduate program	3 years (full-time)	• Endodontics • Paediatric Dentistry • Periodontology • Preventive Dentistry • Prosthodontics • Orthodontics • Oral & Maxillofacial Surgery
On-the-job postgraduate training	3-6 years (part-time)	• Endodontics • Paediatric Dentistry • Periodontology • Preventive Dentistry • Prosthodontics • Orthodontics • Oral & Maxillofacial Surgery
National standardized residency training	3 years (full-time)	• Fields related to Dentistry
Advanced education program Visiting dentists	At least 6 months (full-time)	• Fields related to Dentistry

Other than pursuing clinical training, dental graduates who are interested in research can apply for further study as research postgraduates pursuing Master of Philosophy (MPhil) or Doctor of Philosophy (PhD) degree in the HKU Faculty of Dentistry (16). These MPhil and PhD degrees are not registrable additional dental qualifications because MPhil and PhD students are trained to acquire research skills but not clinical skills. In addition, the enrolment is not limited to dental graduates. However, dental graduates who wish to become academics in HKU or other universities usually have to obtain a PhD degree and relevant advanced clinical training.

### Japan

There are 29 dental colleges and universities in Japan—11 national, 1 public, and 17 private (17). In most cases, students enter dental programs immediately after high school. The students are required to take a six-year curriculum in dental school. When students complete all the requirements of the dental school, they can take a national dental examination. After passing this examination, they will receive a dental license and start residency training in a training facility (hospital or clinic) which is recognized by the Ministry of Health, Labour, and Welfare, where it is necessary to complete at least one year of residency training (mandatory, from 1 April 2006) (18). Before the national dental examination, students have to apply to the dental residency matching program system. The training facilities will make decisions in October during the students’ sixth grade year. The majority of dental students apply through this system, while there are a few students who do not want to join this system. In this case, they are not allowed to practice dentistry or establish their own clinics. They may choose to work in the field of basic science or in public administration department.

Dentists who has graduated and trained outside Japan are required to obtain certificate endorsed by the Minister of Health, Labor and Welfare based on the provisions of the Dental Medical Practitioners’ Act. Upon receiving the approval from the Minister, the dentists are eligible to take the national dental examination (including Japanese practice ability survey). (https://www.mhlw.go.jp/topics/2012/05/tp0525-02.html).

The Ministry of Education, Culture, Sports, Science and Technology Japan reported that the career choice of majority (91.7%) dentists is to conduct clinical work as a general practitioner or dental officer (19). Most dental graduates who have passed the national dental examination will attend residency training at university hospital or medical institution, including national, public, general, and private hospitals, or private dental clinics. With the working experience obtained from the residency training, dental graduates can establish their own dental offices. Some dental graduates choose to practice dentistry employed by a hospital or a dental clinic. Some graduates work for their family clinic with their family members, and to be a successor in charge after the retirement of their family members.

Besides working as a dental practitioner, a few of dental graduates decide to conduct research in a field that they are interested in. They need to pass the entrance examination for a PhD program, which is usually a four-year study period. Publications are commonly required to obtain a PhD degree. In the next step, they may earn a dental specialist’s degree in their field or apply to become a university staff in Japan. Some may also apply for postdoctoral fellows in or outside Japan. In fact, some dental students work as a clinician in dental offices after finishing graduate study and training. Most of them will continue to conduct research or be involved in dental education as a teaching staff in the university. In Japan, only a few dental graduates (less than 8%) wished to be educators or researchers, absolutely a smaller percentage than that in China or Sweden (4, 20). It should be pointed out that compared to other countries, there may be uncertainties in the post-graduation career paths in Japan. Although the postdoctoral system has been improved gradually, it is still not sufficient, and there are only a few openings of teaching positions in universities. Besides, the mandatory residency training system aims to provide dental students clinical training and to help them develop skills to run a dental clinic. It is not surprising that only a small number of dental graduates in Japan wish to start their career in universities as researchers or teaching staff.

Although only a few dental graduates can find a job directly in public health service department after the residency training, it is still possible to apply for this job. Specifically, as civil servants, they can work as technical officials or dental officers in public health service department. A technical official is a technical administrative officer who holds a medical or dental license and plays a central role with their expertise in creating systems related to healthcare. They are members of the Ministry of Health, Labour and Welfare. A dental officer is a senior Self-Defence Force officer involved in the dental care of troops, health management guidance, preventive hygiene, environmental hygiene, and other duties in the health departments of the Ground, Maritime, and Air Self-Defence Forces. There are also other dentists who can obtain civil service-related qualifications and with employment in prefectural or city government offices. There are opportunities of employment in public administration including the Ministry of Health, Labor and Welfare, the Self-Defense Forces, local health centres and government officials.

### Mainland China

Modern dental education in mainland China began in early twentieth century. The first dental school in China was founded in 1917 in Chengdu on the basis of a dental clinic established by Dr Ashely Woodward Lindsay from the University of Toronto (21). Later, the Chinese government recognized dental education as an integral part of medicine, and dental education transformed into a stomatology mode in which faculties of dentistry became schools of stomatology affiliated with medical universities (21). With economic and social development, the Chinese government and the public are paying more and more attention to dental education (22). The dental education mode in mainland China is different from that in western countries (21). In mainland China, the term stomatology, instead of dentistry, is widely used and indicates the traditional philosophy in China that dentistry is a sub-specialty of medicine. Students are admitted to an university majoring in stomatology after graduation from high school (23). There are approximately 100 colleges/universities recruiting students majoring in stomatology with an annual enrolment of around 5000 (24). Students will be awarded a bachelor’s degree in stomatology after completion of a five-year program, which usually consists of four years of didactic and laboratory-based courses and one year of clinical training—a clinical internship. Moreover, an eight-year program is provided by several universities in mainland China where students can obtain doctoral degrees in stomatology (23). After graduation, students are required to gain at least one year of experience in clinical practice in order to be eligible to take the National Physician Qualification Examination specialized in Stomatology (dental licensing qualification examination). The examination consists of two parts, written tests and clinical skills tests. Dental graduates who pass the two parts of the examination will be granted a practicing license which allows them to practice dentistry in mainland China.

As for dental graduates trained outside mainland China, there are special regulations and arrangements regarding the qualification to practice dentistry. As for Hong Kong permanent residents, who have acquired a BDS degree from HKU and who are legally eligible to practice in Hong Kong and have been practicing for more than one year, they are eligible to sit the Mainland’s qualification examination (25). A dental practitioner’s qualification certificate will be issued to those who pass the examination. Despite this, dentists trained outside mainland China can only register and practice dentistry in a limited time period (usually less than 1 year) upon invitation from local healthcare facilities. The registration can be extended and renewed as requested (26).

There are 3 main career choices for dental graduates in mainland China: (1) to be a general dentist, who works mainly in county-level medical institutions or urban private dental clinics; (2) to pursue further training to become a specialist by completing a clinical dental master’s degree program; and (3) to work as teaching or academic staff in a university or college by completing an academic master’s degree program and sometimes a PhD degree is required. It should be pointed out that a national standardized residency training system was established in mainland China in 2014 (27). The national standardized residency training is initiated and funded by the Chinese government. The goal of the program is to improve dental graduates’ practical skills and clinical competence. In the program, dental graduates are required to complete 36 months of clinical training in various disciplines, such as endodontics, prosthodontics, paediatrics and periodontics.

In mainland China, dental care services are provided by both hospital-based institutions and stand-alone clinics. The majority of tertiary and secondary general hospitals provide dental services through their stomatology departments, while major cities have dental or stomatological hospitals, usually affiliated to local dental schools. Specialized dental hospitals, especially those affiliated to regional dental schools, play a key role in providing the majority of dental services in the region, for example, the Peking University Hospital of Stomatology, the Hospital of Stomatology the China Medical University, the Hospital of Stomatology Wuhan University, the West China Hospital of Stomatology Sichuan University, and the Guanghua Hospital of Stomatology Sun Yat-sen University. Although hospitals and public providers offer the majority of dental care services, privately owned dental clinics have been developing rapidly in recent years (28). Besides hospitals and public sectors, dental graduates are attracted to join private dental clinics in residential neighbourhoods with a high population density.

After earning a bachelor’s degree, many dental graduates in mainland China choose to pursue further study in order to specialize in one discipline. About 60 colleges and universities enrol postgraduates majoring in stomatology with an annual enrolment of around 2300, while 40 colleges and universities recruit doctoral students majoring in stomatology with an annual enrolment of around 500. In fact, dental research is considered an important component of dental education in mainland China. Many dental schools in mainland China have claimed to be research oriented, and academic enhancement has long been the developmental strategy of dental schools (29). Research achievement is highly valued in dental schools when recruiting new faculty members. Dental graduates who wish to develop a career as a faculty staff, research achievements will be strictly scrutinized regarding their research achievements and publications.

## Discussion

One of crucial factors for dental graduates to begin their career is the working location (30). In Hong Kong, private dentists concentrated in the central business areas in Kowloon and Hong Kong Island, where the number of residents per private dentist was smaller than 1000. In Japan, dental students need to choose a place for residency training after passing the national dental examination. The majority of dental students choose to have residency training in the university that they graduated from (31). This might be because dental students have seniors and faculty members whom they can consult with, and thus they are less likely to be troubled by human relations and changes in the environment. Thus, they can easily imagine becoming dentists in the near future. On the other hand, some dental students choose to have residency training outside their alma mater in another university hospital or public or private dental hospital. They make their decisions under various considerations, including the income level, the number of patients they can access, and training sessions/seminars they can attend. It should be pointed out that the experience of residency training has a great influence on the choice of career paths for dental graduates, whether pursue further study in graduate school or practicing as a clinician.

Income level remains one of the important considerations for dental graduates when choosing a career path. It is an attractive option for new graduates if they are guaranteed a salary with a steady patient flow. Beyond that, joining public hospitals or large corporate dental practices offers certain benefits that are especially attractive to new dentists, such as continuing education and training opportunities, leadership development programs, vacation and holiday pay, health insurance and professional liability insurance. In mainland China, the government and medical institutions place great importance on continuing education after graduation. Dentists in public hospitals have many opportunities to further develop their knowledge base and improve their clinical skills.

In addition to financial considerations, family obligations and attachment play an important role influencing dental graduates to make choices (4). It is understandable that new graduates choose a familiar environment where family ties make it easier for young graduates to adapt to a new job life. In Japan, some dental students choose to return to their hometown for residency training after graduation. They need to select from candidate training sites in their hometown and apply to a residency training program. However, it may not be possible to fully grasp the personality of the director and staff or the atmosphere of the clinic in only a few days experience.

Life style may be another factor that dental graduates need to take into consideration. In mainland China, dentists in the stomatological department of public general hospitals and dental/stomatological hospitals usually have heavy workloads. With a large number of patients, dentists can gain abundant experience in treating oral diseases and become competent in clinical skills in a relatively short period of time. However, some dental graduates may choose to work in rural areas or small-scale dental clinics with less competition and pressure from peers, so that they can enjoy a balanced work-to-life style.

## Conclusion

Strategies and steps for professional development may vary from place to place as discussed above. In general, dental graduates can choose to be a general dentist working in either private or public sector, pursue further training and education to be a specialist, or work as a researcher or teaching staff in academia. Despite this, a few of dental graduates may join companies, such as dental insurance companies to evaluate claims or dental product incorporations to create and sell dental products, albeit these are not mainstream of career options for dental graduates. Dental graduates will take various factors into consideration when facing career options, which include but not limited to, working location, income level, opportunity for continuing education and training, family obligations and life style.

Besides providing professional training to dental students, it is very important for educators to guide students to find their career paths. Taking different factors into consideration, different choices of career paths are available for dental graduates. They should weigh the pros and cons of different career paths beyond graduation.
